# Xuesaitong Protects Podocytes from Apoptosis in Diabetic Rats through Modulating PTEN-PDK1-Akt-mTOR Pathway

**DOI:** 10.1155/2020/9309768

**Published:** 2020-01-16

**Authors:** Rui Xue, Ruonan Zhai, Ling Xie, Zening Zheng, Guihua Jian, Teng Chen, Jun Su, Chongting Gao, Niansong Wang, Xifei Yang, Youhua Xu, Dingkun Gui

**Affiliations:** ^1^Department of Nephrology, Shanghai Jiao Tong University Affiliated to Sixth People's Hospital, Shanghai 200233, China; ^2^Shanghai Ocean University, Shanghai 201306, China; ^3^Guangzhou University of Traditional Chinese Medicine, Guangzhou 510405, China; ^4^Shanghai University of Traditional Chinese Medicine, Shanghai 201203, China; ^5^Key Laboratory of Modern Toxicology of Shenzhen, Shenzhen Center for Disease Control and Prevention, Shenzhen 518055, China; ^6^Faculty of Chinese Medicine, State Key Laboratory of Quality Research in Chinese Medicine, Macau University of Science and Technology, Taipa, Macao 999078, China

## Abstract

Diabetic kidney disease (DKD) is a major cause of end-stage renal disease (ESRD), and therapeutic strategies for delaying its progression are limited. Loss of podocytes by apoptosis characterizes the early stages of DKD. To identify novel therapeutic options, we investigated the effects of Xuesaitong (XST), consisting of total saponins from Panax notoginseng, on podocyte apoptosis in streptozotocin- (STZ-) induced diabetic rats. XST (5 mg/kg·d) or Losartan (10 mg/kg·d) was given to diabetic rats for 12 weeks. Albuminuria, renal function markers, and renal histopathology morphological changes were examined. Podocyte apoptosis was determined by triple immunofluorescence labelling including a TUNEL assay, WT1, and DAPI. Renal expression of Nox4, miRNA-214, PTEN, PDK1, phosphorylated Akt, mTOR, and mTORC1 was detected. In diabetic rats, severe hyperglycaemia and albuminuria developed, and apoptotic podocytes were markedly increased in diabetic kidneys. However, XST attenuated albuminuria, mesangial expansion, podocyte apoptosis, and morphological changes of podocytes in diabetic rats. Decreased expression of PTEN, as well as increased expression of Nox4, miRNA-214, PDK1, phosphorylated Akt, mTOR, and mTORC1, was detected. These abnormalities were partially restored by XST treatment. Thus, XST ameliorated podocyte apoptosis partly through modulating the PTEN-PDK1-Akt-mTOR pathway. These novel findings might point the way to a natural therapeutic strategy for treating DKD.

## 1. Introduction

Diabetic kidney disease (DKD), one of the most common microvascular complication of diabetes, is a serious public health concern in the developed countries [1]. Thus, therapeutic strategies for preventing or delaying the progression of DKD are urgently needed. Podocyte injury is one of the pathological changes throughout the progression of DKD [2]. The reduction of the podocyte number contributes to the progression, and impaired podocyte function acts as a trigger to accelerate kidney function decline [3, 4]. Moreover, expression of podocyte marker proteins, such as synaptopodin, podocin, and nephrin, was significantly decreased, which could result in podocyte cytoskeleton disorder, damaged sufficient adhesion, and separation between podocyte and the GBM in DKD [5]. Recent studies further demonstrated the crucial role of apoptosis in the reduction of the podocyte number at the early stage of DKD [6–8]. It has been reported that high glucose caused apoptosis of podocytes both in vivo and in vitro [9]. Thus, podocyte apoptosis and subsequent podocyte depopulation are a crucial triggering mechanism leading to DKD [7]. This novel discovery may offer promising insights to develop new pharmacological interventions for preventing DKD. Panax notoginseng, an edible Chinese herb, has been included in the list of health Chinese herbs with drug and food properties announced by the National Health Commission of China. Xuesaitong (XST) is a traditional Chinese medical compound consisting of total saponins from Panax notoginseng. XST could effectively treat the patients with cerebrovascular diseases [10]. XST attenuated ischemic stroke in mice through regulating microglial phenotypes and decreasing apoptosis of neuronal cells [11]. XST also ameliorated ischemia-reperfusion-induced myocardial [12] and intestinal [13] damage in rats. Recent study demonstrated that Panax notoginseng saponins (PNS), the major active ingredients of XST, protected rat retinal capillary endothelial cells from oxidative injury induced by high glucose [14]. However, the protective effects of XST on podocyte apoptosis in DKD have not been sufficiently investigated. Therefore, this study is aimed at examining the effects of XST on podocyte apoptosis and then points the way to a natural therapeutic strategy in DKD.

## 2. Materials and Methods

### 2.1. Materials

A Xuesaitong (XST) dispersible tablet was obtained from Yige Pharmaceutical Co. Ltd. (Xiangtan, Hunan, China). Streptozotocin (STZ) was obtained from Sigma-Aldrich company (Sigma-Aldrich, USA). Antibodies of PTEN, PDK1, Nox4, nephrin, and 4′,6-diamidino-2-phenylindole (DAPI) were purchased from Abcam (Cambridge, UK); antibodies of Akt, p-Akt, mTOR, and p-mTOR were acquired from Cell Signaling Technology (Danvers, MA, USA). WT1, GAPDH, and *β*-Tubulin were purchased from Absin Bioscience Inc. (Shanghai, China). Antibodies of horseradish peroxidase-conjugated rabbit and mouse IgG were obtained from Beyotime Institute of Biotechnology (Shanghai, China). The assay kit for terminal deoxynucleotidyl transferase-mediated dUTP nick end labelling (TUNEL) was obtained from Shanghai Yisheng Chemical Company Limited (Shanghai, China). The assay kit for bicinchoninic acid (BCA) protein was obtained from Biosharp Biotechnology Company Limited.

### 2.2. Animal Studies

The experimental protocols of this study were approved by the Animal Ethics Committee of Shanghai Sixth People's Hospital. All animal experiments followed the National Institutes of Health Guide. Healthy male Sprague-Dawley (SD) rats, weighing 180-200 g, were placed in specific pathogen-free (SPF) environment. The diabetes model was induced by intraperitoneal injection of STZ at a dose of 55 mg/kg. The blood glucose level was detected from the tail vein after injection of STZ. Rats with a blood glucose level exceeding 16.7 mmol/L were considered to be diabetic rats. The diabetic rats were then randomly divided into three groups (*n* = 8 each group): diabetic rats only receiving normal saline (model), diabetic rats receiving XST at 5 mg/kg·d (XST), and diabetic rats receiving Losartan at 10 mg/kg·d (Losartan). None of the diabetic rats were orally given equal volume of normal saline (normal, *n* = 8). After blood glucose reached beyond 16.7 mmol/L, XST or Losartan was intragastrically administrated to rats once daily for 12 weeks. Metabolic cages were placed for collection of 24 h urine of rats. The urinary albumin/creatinine ratio (ACR) was measured by a biochemistry analyser (HITACHI 7600-120E, Japan). At the end of 12 weeks of treatment, all the rats were sacrificed. Blood samples were taken, and kidney samples were harvested quickly.

### 2.3. Renal Histological Analysis

Kidney tissues were embedded in paraffin for morphological analysis. Renal tissues were cut into 4 *μ*m sections. Hematoxylin-Eosin (HE) and periodic acid-Schiff (PAS) staining was carried out on the serial kidney sections. The sections were then examined under light microscopy (Leica, Germany). The kidney cortex was also fixed with 2% glutaraldehyde for 2 h at 4°C. Uranyl acetate and lead citrate staining was performed to examine the morphological changes under electron microscopy (Philip CM-120, Netherlands). The histological evaluation was performed by two blinded investigators.

### 2.4. Immunohistochemical Staining

Immunohistochemical staining was performed on 4 *μ*m paraffin-embedded renal sections after a descending ethanol gradient of dewaxing. Antibodies of PTEN, PDK1, Nox4, nephrin, *α*-dystroglycan, p-Akt, and p-mTOR were diluted in PBS and then incubated overnight at 4°C. These sections incubated with TBST were chosen as negative controls. After incubation with horseradish peroxidase- (HRP-) conjugated anti-rabbit and anti-mouse IgG antibodies for 1 hour at 37°C, the sections were visualized by DAB solution. The pathological ImageJ software analysis system (Adobe Corp., USA) was used for quantitative analysis. All slides were analysed by two investigators in a blinded manner.

### 2.5. Immunofluorescence and TUNEL Assay

Podocyte apoptosis was examined by triple immunofluorescence labelling including the terminal deoxynucleotidyl transferase-mediated dUTP nick end labelling (TUNEL) assay, WT1, and 4′,6-diamidino-2-phenylindole (DAPI) on renal frozen sections. The cells with WT1 (red), TUNEL (green), and DAPI (blue) were considered as the positive apoptotic podocytes. The WT1- and DAPI-positive but TUNEL-negative cells were considered as negative apoptotic podocytes. The images were examined by a laser confocal microscope (Leica, Germany). The sections were evaluated independently by two investigators in a blinded manner.

### 2.6. Western Blotting

Renal tissues were homogenized on ice with a homogenizer. The BCA protein assay was used to determine the protein concentration. The protein from kidney tissue was separated by using SDS polyacrylamide gel electrophoresis and then transferred onto a PVDF membrane (Millipore, USA), then blocked with 5% BSA for 1 h at 37°C and then incubated with antibodies for PTEN, PDK1, Nox4, nephrin, *α*-dystroglycan, Akt, p-Akt, mTOR, p-mTOR, and mTORC1 at 37°C overnight. The membrane was incubated with horseradish peroxidase-labelled secondary antibody for 1 h at 37°C. The bands were visualized by a Pierce ECL Super Signal kit (Thermo, Waltham, MA, USA). Protein expression was quantitatively analysed by the ratio of marker bands to housekeeping bands.

### 2.7. Real-Time Quantitative PCR

Total RNA was isolated by using a TRIzol reagent (Invitrogen, Carlsbad, CA) and then reverse-transcribed into cDNA. The sequence-specific primers were described as follows: rno-miRNA-214: 5′GGGGAACAGCAGGCACAGA3′ (GSP) and 5′ GTGCGTGTCGTGGAGTC G3′ (reverse); U6: 5′GCTTCGGCAGCACATATACTAAAAT3′ (forward) and 5′ CGCTTCACGAATTTGCGTGTC AT3′ (reverse). Quantitative PCR was performed by the ABI PRISM 7900 Sequence Detection System (Applied Biosystems, Carlsbad, CA). The relative changes in gene expression were quantitatively analysed by the ratio of specific gene to U6 mRNA level.

### 2.8. Statistical Analysis

SPSS 18.0 software was used to perform statistical analysis. Data were expressed as means ± standard error and analysed by one-way analysis of variance. *P* < 0.05 was considered as statistically significant.

## 3. Results

### 3.1. Effects of XST on Physical and Biochemical Parameters in Diabetic Rats

**Figure 1:**
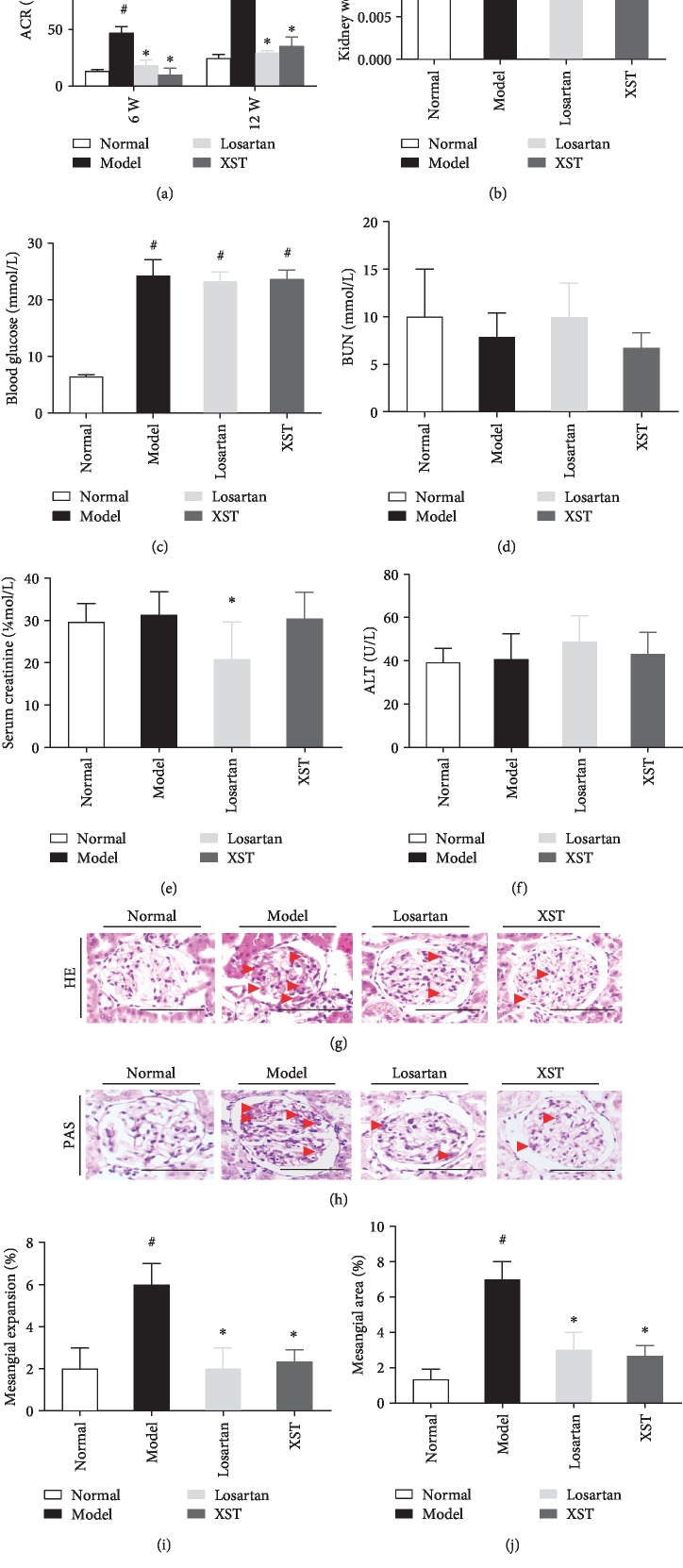
Effects of XST on physical and biochemical parameters, as well as renal histopathology in diabetic rats. Effects of XST on urinary ACR (a), kidney weight/body weight ratio (b), BUN (d), serum creatinine (e), and ALT (f) in diabetic rats. Blood glucose at baseline in rats (c). Representative HE (g) and PAS (h) staining of kidney sections. Representative quantitative analysis for mesangial matrix expansion (i, j). Normal: normal control rats; model: STZ-induced diabetic rats; Losartan: diabetic rats treated with Losartan; XST: diabetic rats treated with Xuesaitong. XST (5 mg/kg·d) or Losartan (10 mg/kg·d) was intragastrically administrated once daily for 12 weeks. ACR: albumin/creatinine ratio; BUN: blood urea nitrogen; ALT: alanine aminotransferase. Scale bar = 100 *μ*m. Red arrowheads indicated mesangial expansions. Results were expressed as the means ± SEM (*n* = 8). ^#^*P* < 0.05 vs. normal; ^∗^*P* < 0.05 vs. model.

Severe hyperglycaemia and albuminuria developed in diabetic rats. XST or Losartan significantly decreased urinary ACR in diabetic rats after 6 and 12 weeks of treatment. However, XST caused a greater reduction in the ACR level than did Losartan after 6 weeks of treatment (Figure 1(a)). Thus, XST treatment attenuated albuminuria in diabetic rats. XST reduced the ratio of kidney weight to body weight in diabetic rats when compared to the normal control (Figure 1(b)). Blood glucose (BG) was significantly increased in diabetic rats when compared to the normal control (Figure 1(c)). However, there were no significant differences in blood urea nitrogen (BUN) (Figure 1(d)), creatinine (Cr) (Figure 1(e)), and alanine aminotransferase (ALT) (Figure 1(f)) between XST-treated and untreated diabetic rats. These results demonstrated that XST had no apparent toxicity to the kidney and liver.

### 3.2. Effects of XST on Renal Mesangial Matrix Expansion and Morphological Changes of Podocytes in Diabetic Kidneys

**Figure 2:**
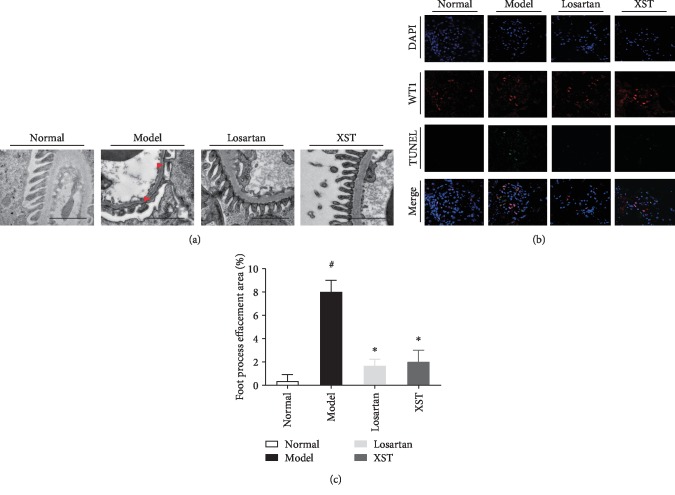
Effects of XST on podocyte morphological changes and podocyte apoptosis in diabetic rats. Representative transmission electron photomicrographs (a) and semiquantitative ultrastructural analysis (c) in diabetic rats. Representative immunofluorescence labelling including the TUNEL assay, WT1, and DAPI (b) on frozen kidney sections. The cells with WT1 (red), TUNEL (green), and DAPI (blue) were identified as the positive apoptotic podocytes. Scale bar = 2 *μ*m. Red arrowheads indicated podocyte foot process effacement. Results were expressed as the means ± SEM. ^#^*P* < 0.05 vs. normal; ^∗^*P* < 0.05 vs. model.

As shown in Figure 1, the diabetic rats have an increased mesangial area. However, XST significantly attenuated mesangial expansion compared with model diabetic rats (Figures 1(g) and 1(h)). Quantification of renal histopathology exhibited that the mesangial area was significantly elevated in model rats, but XST treatment markedly decreased the mesangial area compared to the model rats (Figures 1(i) and 1(j)). Moreover, conspicuous podocyte foot process effacement was observed in model rats when compared to controls, while the XST-treated rats showed an ameliorative podocyte foot process (Figure 2(a)). Semiquantitative ultrastructural analysis further indicated that XST also attenuated podocyte foot process effacement in diabetic rats (Figure 2(c)). These results showed that XST ameliorated renal histopathology and restored podocyte morphology in diabetic rats.

### 3.3. Effects of XST on Podocyte Apoptosis in Diabetic Kidneys

Immunofluorescence labelling including the TUNEL assay, WT1, and DAPI on renal frozen sections was performed to accurately detect the protective effects of XST against podocyte apoptosis. The diabetic rats underwent podocyte apoptosis, while the XST group showed a significant decrease in podocyte apoptosis (Figure 2(b)). The podocyte number was decreased in diabetic rats, while the rats treated with XST showed an apparent increase in the podocyte number (Figure 2(b)). Thus, XST prevented podocyte apoptosis and podocyte detachment in diabetic rats.

### 3.4. Effects of XST on Expression of *α*-Dystroglycan and Nephrin in Diabetic Kidneys

**Figure 3:**
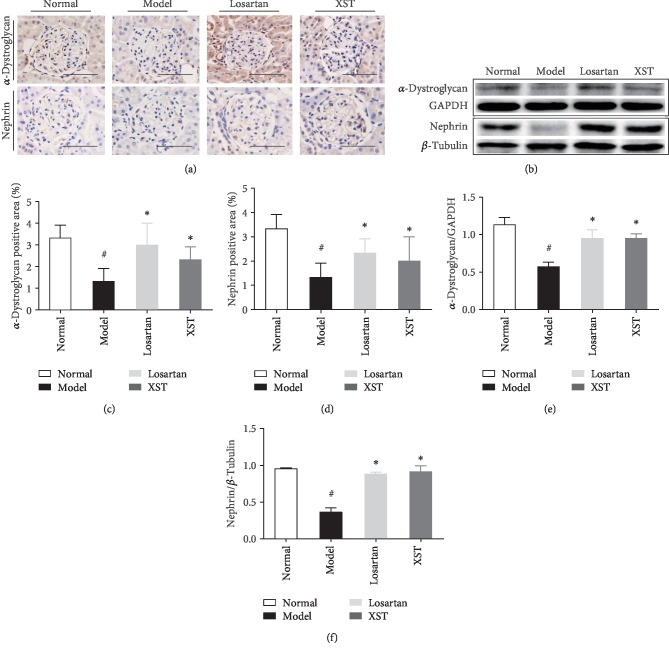
Effects of XST on expression of *α*-dystroglycan and nephrin in diabetic kidneys. Representative immunohistochemical staining (a) and western blotting (b) for *α*-dystroglycan and nephrin in the glomerulus. Quantitative analysis for *α*-dystroglycan and nephrin expression (c–f). Results were expressed as the means ± SEM. Scale bar = 100 *μ*m. ^#^*P* < 0.05 vs. normal; ^∗^*P* < 0.05 vs. model.

The level of nephrin and *α*-dystroglycan was significantly decreased in diabetic rats compared to controls. However, XST treatment restored the level of nephrin and *α*-dystroglycan in diabetic rats (Figure 3(a)). Quantitative analysis also showed that XST elevated the renal expression of *α*-dystroglycan and nephrin in diabetic rats (Figures 3(c) and 3(d)). As shown in western blot analysis, the protein level of nephrin and *α*-dystroglycan was significantly decreased in diabetic kidneys. However, XST markedly restored *α*-dystroglycan and nephrin expression in diabetic kidneys (Figure 3(b)). Quantitative analysis also demonstrated that XST restored the level of *α*-dystroglycan and nephrin in diabetic rats (Figures 3(e) and 3(f)). Thus, XST restored the level of *α*-dystroglycan and nephrin, thus ameliorating podocyte detachment.

### 3.5. Effects of XST on Expression of Nox4, miRNA-214, PTEN, and PDK1 in Diabetic Rats

**Figure 4:**
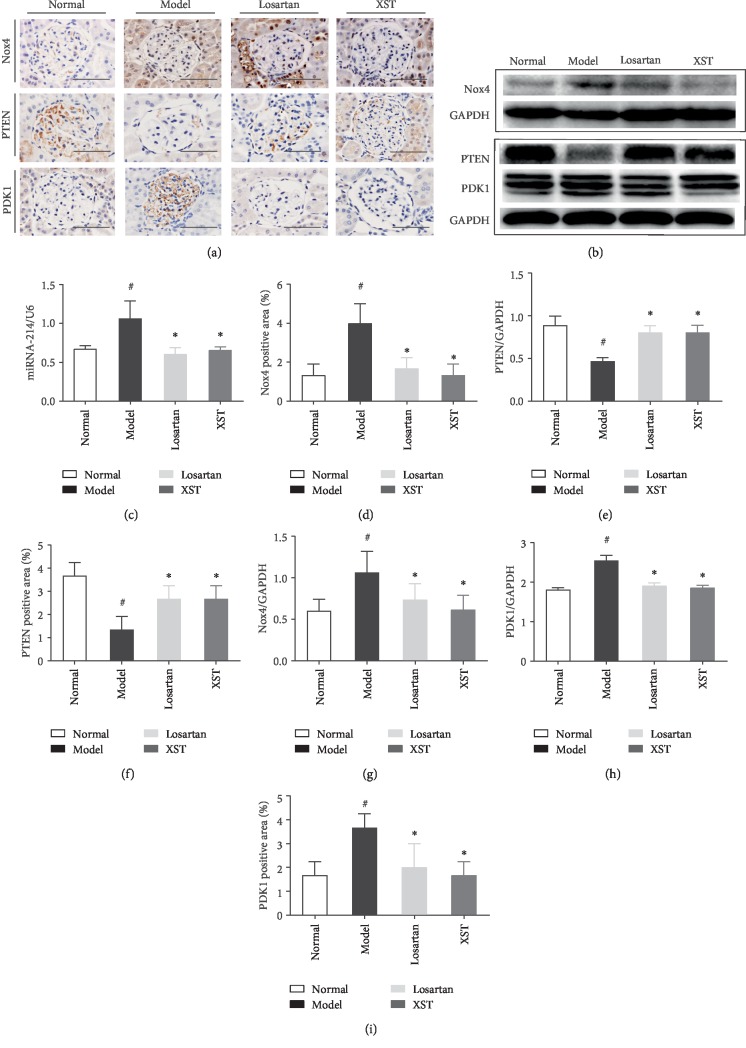
Effects of XST on Nox4, miRNA-214, PTEN, and PDK1 expression in diabetic rats. Representative immunohistochemical staining (a) and western blotting (b) for Nox4, PTEN, and PDK1 in glomerulus. Representative real-time quantitative PCR for miRNA-214 (c). Quantitative analysis for Nox4, PTEN, and PDK1 (d–i). Scale bar = 100 *μ*m. ^#^*P* < 0.05 vs. normal; ^∗^*P* < 0.05 vs. model.

Nox4 expression was markedly elevated in diabetic rats; however, XST reduced the positive area of Nox4 expression in diabetic rats (Figures 4(a) and 4(d)). As shown in western blot analysis, Nox4 expression was significantly elevated in diabetic kidneys, which was partially restored by XST (Figures 4(b) and 4(g)). The miR-214 mRNA expression was elevated in STZ-induced diabetic rats. However, XST significantly reduced the miR-214 mRNA expression in diabetic glomeruli (Figure 4(c)). Thus, XST protected against podocyte apoptosis partly by downregulation of Nox4 and miR-214 expression induced by hyperglycaemia. PTEN expression was markedly decreased in diabetic kidneys while XST partly restored PTEN expression (Figures 4(a) and 4(f)). Moreover, PDK1 expression was significantly increased in diabetic kidneys. However, XST treatment reduced PDK1 expression in diabetic rats (Figures 4(a) and 4(i)). These results were further supported by the findings of western blot analysis. The protein expression of PTEN was reduced in diabetic rats while XST increased protein production of PTEN (Figures 4(b) and 4(e)). PDK1 expression was markedly elevated in diabetic kidneys while XST treatment could reduce PDK1 expression in diabetic rats (Figures 4(b) and 4(h)).

### 3.6. Effects of XST on Phosphorylated Akt, mTOR, and mTORC1 Expression in Diabetic Rats

**Figure 5:**
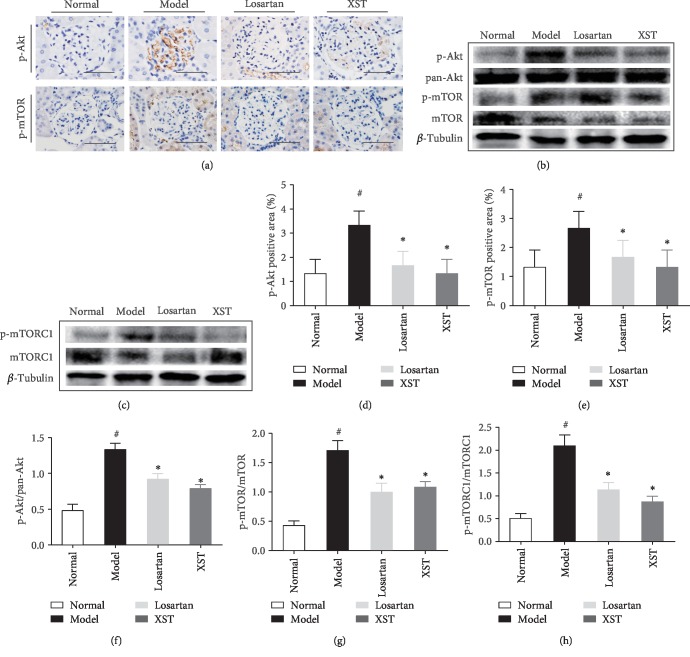
Effects of XST on p-Akt, p-mTOR, and p-mTORC1 expression in diabetic kidneys. Representative immunohistochemical staining (a) and western blotting (b, c) for p-Akt, p-mTOR, and p-mTORC1 in glomerulus. Quantitative analysis for p-Akt, p-mTOR, and p-mTORC1 (d–h). Scale bar = 100 *μ*m. Results were expressed as the means ± SEM. ^#^*P* < 0.05 vs. normal; ^∗^*P* < 0.05 vs. model.

Immunohistochemical staining revealed that phosphorylated Akt (p-Akt) and mTOR (p-mTOR) expression was increased in diabetic kidneys. However, XST significantly reduced p-Akt and p-mTOR expression in diabetic kidneys (Figures 5(a), 5(d), and 5(e)). Western blot analysis further indicated that p-Akt, p-mTOR, and mTORC1 (p-mTORC1) were significantly increased in diabetic rats while XST markedly inhibited the activation of p-Akt, p-mTOR, and p-mTORC1 (Figures 5(b), 5(c), and 5(f)–5(h)). These findings demonstrated that regulation of the PTEN-PDK1-Akt-mTOR signalling pathway was involved in the antiapoptotic effect of XST on podocytes.

## 4. Discussion

DKD has become a global health problem; however, the therapeutic strategies for preventing its progression are limited. XST is a traditional Chinese medical compound consisting of the total saponins extracted from Panax notoginseng. The novel finding of this work was the discovery of a natural product for treatment of DKD. We firstly reported that XST protected against podocyte apoptosis partly by regulation of the PTEN-PDK1-Akt-mTOR pathway. Our conclusion was based on the following evidence: (i) XST significantly ameliorated albuminuria, mesangial expansion, podocyte apoptosis, and podocyte morphological changes in diabetic kidneys; (ii) XST partially restored PTEN expression, as well as apparently inhibited PDK1 expression and activations of phosphorylated Akt, mTOR, and mTORC1 in diabetic kidneys; and (iii) XST markedly decreased expression of miR-214, the upstream regulator of the PTEN-PDK1-Akt-mTOR pathway. Losartan, the current first-line therapy of DKD, was chosen as a positive control to investigate the protective effects of XST on albuminuria in diabetic rats. In agreement with our previous reports [9, 15], the diabetic rats developed severe albuminuria. However, XST decreased the ACR level to a greater degree than did Losartan after 6 weeks of treatment. Therefore, XST could be developed into a novel drug for clinical treatment of DKD.

Loss of podocytes by apoptosis characterizes the early stages of DKD. To identify new therapeutic strategies, we firstly examined the protective effects of XST against podocyte apoptosis in diabetic kidneys. WT1, a specific marker for podocyte, is greatly expressed in podocyte nuclei [16]. In this study, the immunofluorescence labelling of TUNEL, WT1, and DAPI in frozen sections was performed to accurately detect podocyte apoptosis. We found that XST significantly inhibited podocyte apoptosis in diabetic kidneys.

To explore the underlying mechanisms of XST for protecting against podocyte apoptosis, we examined the effects of XST on the PTEN-PDK1-Akt-mTOR pathway in diabetic rats. PTEN regulates cell growth, migration, metabolism, and so on [17, 18]. Loss of PTEN induces podocyte cytoskeletal rearrangement and also leads to the development of proteinuria and DKD [19]. Podocyte-specific knock in of PTEN protected against kidney injury in a diabetic mouse model [20]. PDK1 can promote cell proliferation through regulation of the Akt pathway [21, 22]. PTEN, one of the upstream negative regulators of phosphatidylinositol 3 kinase- (PI3K-) PDK1 signalling, inhibited follicular activation [23]. It has been reported that PI3K/PTEN-PDK1 signalling in oocytes regulates the survival, loss, and activation of primordial follicles [24]. PDK1 is one of the activators of the Akt cell survival pathway and might be associated with the development of DKD through regulation of podocyte apoptosis [25, 26]. The PDK1/Akt/mTOR pathway was activated both in diabetic rats and in human renal mesangial cells under high glucose condition [27]. Previous study provided evidence for mTOR's novel function on podocyte apoptosis in DKD [5], and mTOR inhibition could protect against podocyte apoptosis and delay the progression of DKD [28]. The mTOR complexes play an important role in cancer metabolism [29]. Moreover, mTORC1 activation in podocytes played a crucial role in the development of DKD [30]. Taken together, the PTEN-PDK1-Akt-mTOR pathway is closely associated with podocyte apoptosis in DKD. In this study, XST restored PTEN expression, inhibited the PDK1/Akt/mTOR pathway, and phosphorylated mTORC1 activation in diabetic kidneys. Thus, the underlying mechanisms of XST for protecting against podocyte apoptosis were associated with the regulation of the PTEN-PDK1-Akt-mTOR pathway.

We next investigated the effects of XST on the upstream regulator of the PTEN-PDK1-Akt-mTOR pathway. Our previous study showed that PTEN was the target of miR-214 [31]. Inhibition of miR-214 significantly restored PTEN expression and attenuated albuminuria and mesangial expansion in db/db mice [31]. It was reported that miR-214-targeted PTEN contributed to Akt activation, which resulted in mTORC1 activation [32]. The above studies indicated that miR-214 was an upstream regulator of the PTEN-Akt-mTOR pathway. Here, XST inhibited miR-214 expression and PDK1/Akt/mTOR pathway in diabetic kidneys. Therefore, the regulatory effects of XST on miR-214 might be accountable for its action on the PTEN-PDK1-Akt-mTOR pathway and apoptosis of podocytes in diabetic kidneys.

High glucose induces podocyte apoptosis by excessive reactive oxygen species (ROS) production through upregulation of Nox4 [6]. It has been demonstrated that Nox4 expression is increased in diabetic kidneys [33]. Recent study reported that inhibition of Nox4 attenuated albuminuria in a mouse model of DKD [34]. Oxidant stress played an important role in the pathophysiology of diabetic microvascular complications [35]. The protective effects of targeting Nox4 have been demonstrated in streptozotocin-induced diabetic mice, where Nox4 knockout was associated with prevention of glomerular damage. Podocytes express mainly Nox1, 4, and 5 and p22phox while Nox1, 2, 4, and 5 and p47phox have been identified in glomerular endothelial cells [36, 37]. Nox5 is also expressed in proximal tubular cells, with Nox4 and Nox1 distributed in the whole tubular compartment [38].

Our study showed that XST reduced expression of Nox4, responsible for ROS production. It will be important to show that XST reduced ROS markers such as 8-OHdG and 4-hydroxy-2-nonenal. Our study demonstrated that XST decreased Nox4 expression and podocyte apoptosis in diabetic kidneys. We added the immunohistochemical staining for 8-OHdG and 4-hydroxy-2-nonenalin in kidneys. We demonstrated that 8-OHdG and 4-hydroxy-2-nonenalin expression was markedly elevated in diabetic kidneys; however, XST reduced the expression of ROS markers such as 8-OHdG and 4-hydroxy-2-nonenal in kidneys (Supplementary [Supplementary-material supplementary-material-1]). We also showed that XST reduced the p-Akt pathway, which is potentially involved in podocyte apoptosis induced by hyperglycaemia. We also reconciled the role of Akt and ROS in these processes. Nephrin is associated with the maintenance of the normal podocyte actin cytoskeleton structure and function through interactions with signalling proteins and cascades, namely, PI3K-dependent protein kinase B (Akt) [39]. Thus, the Akt phosphorylation may cause the podocyte injuries by reducing the podocyte-related proteins in DKD. Akt promotes mitochondria oxygen consumption and contributes to ROS accumulation [40, 41]. Moreover, Akt promotes oxygen consumption and increases ROS production induced by hyperglycaemia through activating mTORC1 [42]. Our study showed that the PTEN-PDK1-Akt-mTOR pathway played a crucial role in DKD, and the relationship between ROS and PTEN-PDK1-Akt-mTOR pathways is closely linked. Thus, Akt could regulate ROS in DKD. We found that XST exerted antioxidant antiapoptotic effects through PTEN-PDK1-Akt-mTOR signalling pathways. Furthermore, the inhibited effect of XST on podocyte apoptosis may be associated with inhibition of Nox4 expression and reduction of oxidative stress. Taken together, XST acted on the oxidative stress and apoptosis of podocytes. This will be an important point to elucidate in order to validate the use of XST as a novel therapeutic drug for DKD and other kidney diseases affecting podocytes.

XST also restored the protein expression of nephrin, a key structural component of the slit diaphragm, and *α*-dystroglycan, one of the important cell-matrix adhesion receptors expressed in podocytes, and then ameliorated podocyte detachment. Therefore, XST also attenuated podocyte detachment and podocyte depletion in diabetic kidneys. Panax notoginseng saponins (PNS) are the major effective constituents of XST. The main components of PNS included Ginsenoside Re, Ginsenoside Rb1, Notoginsenoside R1, Ginsenoside Rg1, and Ginsenoside Rd, which were determined by HPLC [43]. Our previous report showed that Notoginsenoside R1 ameliorated podocyte detachment in diabetic rats [44]. Thus, Notoginsenoside R1 might be one of the main components of XST. Moreover, XST did not change the level of BUN, Cr, and ALT, which indicated that XST had no apparent toxicity to the kidney and liver. These findings suggested that XST might have considerable safety.

## 5. Conclusions

**Figure 6:**
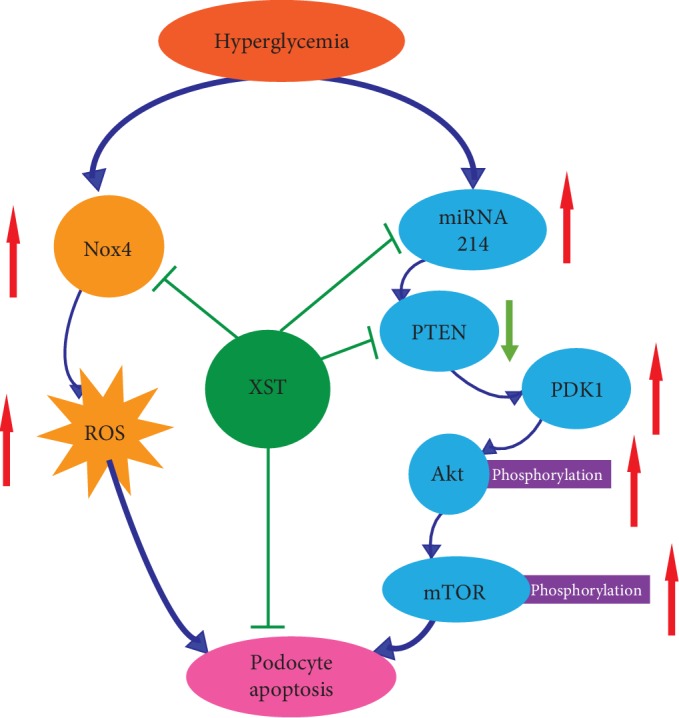
Graphic representation of the mechanism of Xuesaitong (XST) against podocyte apoptosis partly through modulation of the PTEN-PDK1-Akt-mTOR pathway and Nox4 expression.

XST ameliorates podocyte apoptosis in diabetic rats partly through modulation of the PTEN-PDK1-Akt-mTOR pathway and Nox4 expression (Figure 6). These findings might point the way to a novel natural therapy for DKD.

## Data Availability

The data that support the findings of this study are available from the corresponding authors upon reasonable request.

## Supplementary Materials

Supplementary Materials Effects of XST on 8-OHdG and 4-hydroxy-2-nonenalin expression in diabetic kidneys. Representative immunohistochemical staining for 8-OHdG (A) and 4-hydroxy-2-nonenalin (B) in glomerulus. Quantitative analysis for 8-OHdG (C) and 4-hydrox y-2-nonenalin (D). Scale bar = 100 *μ*m. ^#^*P* < 0.05 vs. normal; ^∗^*P* < 0.05 vs. model.
