# Supplementary material for: Xuesaitong Protects Podocytes from Apoptosis in Diabetic Rats through Modulating PTEN-PDK1-Akt-mTOR Pathway
Source: J Diabetes Res. 2020 Jan 16;2020:9309768. doi: 10.1155/2020/9309768 (PMC6995497; doi:10.1155/2020/9309768)
Supplement: Supplementary Materials — Effects of XST on 8-OHdG and 4-hydroxy-2-nonenalin expression in diabetic kidneys. Representative immunohistochemical staining for 8-OHdG (A) and 4-hydroxy-2-nonenalin (B) in glomerulus. Quantitative analysis for 8-OHdG (C) and 4-hydrox y-2-nonenalin (D). Scale bar = 100 μm. #P < 0.05 vs. normal; ∗P < 0.05 vs. model. [file 9309768.f1.docx]

**Supplementary Materials**

# Journal of Diabetes Research

# Xuesaitongprotectspodocytes from apoptosis in diabetic rats through modulating PTEN-PDK1-Akt-mTOR pathway

Rui Xue^1^, Ruonan Zhai^1^, Ling Xie^2^, Zening Zheng^3^, Guihua Jian^1^, Teng Chen^4^, Jun Su^1^, Chongting Gao^1^, Niansong Wang^1^, Xifei Yang^5^,Youhua Xu^6^, Dingkun Gui^1^

^1^Department of Nephrology, Shanghai Jiao Tong University Affiliated Sixth People’s Hospital, Shanghai,200233,China.

^2^Shanghai Ocean University, Shanghai, 201306, China.

^3^Guangzhou University of Traditional Chinese Medicine, Guangzhou, 510405, China.

^4^Shanghai University of Traditional Chinese Medicine, Shanghai, 201203, China.

^5^Key Laboratory of Modern Toxicology of Shenzhen, Shenzhen Center for Disease Control and Prevention, Shenzhen, 518055, China.

^6^Faculty of Chinese Medicine, State Key Laboratory of Quality Research in Chinese Medicine,Macau University of Science and Technology, Taipa, Macao, 999078, China.

Correspondence should be addressed to DingkunGui; dingkungui@alu.fudan.edu.cn, Xifei Yang; xifeiyang@gmail.com andYouhua Xu; yhxu@must.edu.mo

Excessive production of reactive oxygen species (ROS) is widely recognized as a critical factor leading to podocyte apoptosis induced by hyperglycaemia. 8-hydroxy-2'-deoxyguanosine (8-OHdG) and 4-hydroxy-2-nonenalin are repair products of oxidized guanine lesions and have been acknowledged as biomarkers of oxidative stress. Here, we added the immunohistochemical staining of ROS markers such as 8-OHdG and the 4-hydroxy-2-nonenalin to examine the effects of on expression of ROS markers in kidney.

**Materials and Methods**

**Immunohistochemical staining**

Immunohistochemical staining were performed 4μm paraffin-embedded renal sections after a descending ethanol gradient of dewaxing. Antibodies of 8-OHdG (N45.1) and the 4-hydroxy-2-nonenalin (HNEJ-2) were 1:200 diluted in PBS and then incubated overnight at 4°C. These sections incubated with TBST were chosen as negative controls. After incubation with horseradish peroxidase (HRP) conjugated anti-rabbit and anti-mouse IgG antibodies for 1 hour at 37°C, the sections were visualized by DAB solution. Pathological Image J software analysis system (Adobe Corp, USA) was used for quantitative analysis. All slides were analysed by two unaware investigators in a blinded manner.

**Results**

**Effects of XST on expression of****8-OHdG and the** **4-hydroxy-2-nonenalinin diabetic rats**

We added the immunohistochemical staining for 8-OHdG and 4-hydroxy-2-nonenalin in kidneys. We demonstrated that 8-OHdG and 4-hydroxy-2-n

onenalin expression were markedly elevated in diabetic kidneys, however, XST reduced the expression of ROS markers such as 8-OHdG and 4-hydroxy-2-nonenal in kidneys. (Supplementary Figure 1).


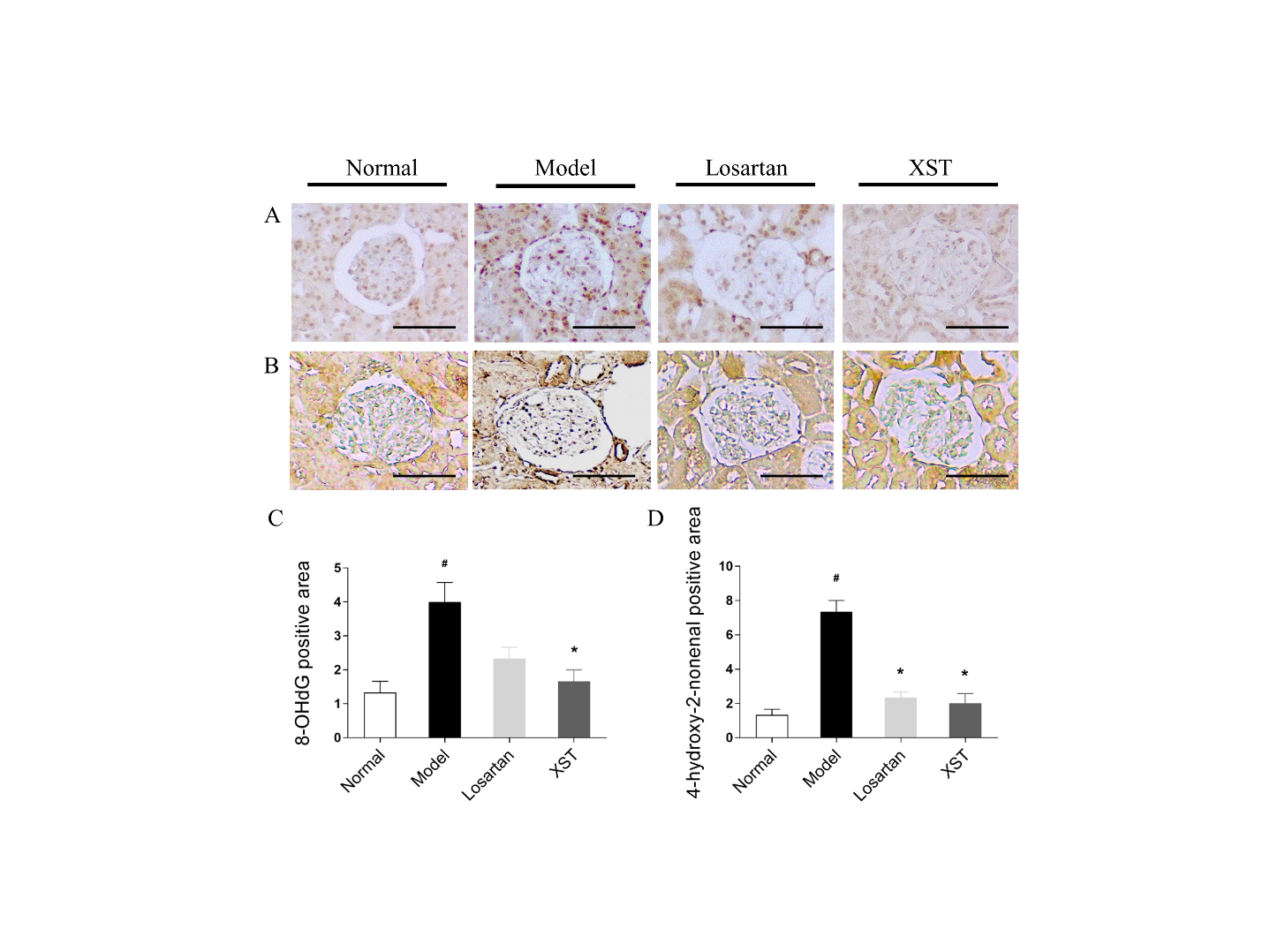


**Supplementary Figure 1.** Effects of XST on 8-OHdG and 4-hydroxy-2-nonenalin expression in diabetic kidneys. Representative immunohistochemical staining for 8-OHdG (A) and 4-hydroxy-2-nonenalin (B) in glomerulus. Quantitative analysis for 8-OHdG (C) and 4-hydrox y-2-nonenalin (D). Scale bar = 100 μm.  ^#^*P*<0.05 vs Normal; **P*<0.05 vs Model.
